# An acute gastric volvulus in a child with congenital left diaphragmatic hernia: a case report

**DOI:** 10.1186/s12887-024-04834-8

**Published:** 2024-05-20

**Authors:** Zesheng Yang, Xiaoying Xie, Shicheng Wang, Guanghua Pei, Jianghua Zhan

**Affiliations:** 1 Tianjin Children’s Hospital (Children’s Hospital of Tianjin University), Tianjin, China; 2 Department of Ultrasound, Tianjin Children’s Hospital (Children’s Hospital of Tianjin University), No.238 Longyan Road, Beichen District, Tianjin, 300134 China; 3 Department of General Surgery, Tianjin Children’s Hospital(Children’s Hospital of Tianjin University), Tianjin, China

**Keywords:** Ultrasound, Whirlpool sign, Children, Acute gastric volvulus

## Abstract

**Background:**

Acute complete gastric volvulus is a rare and life-threatening disease, which is prone to gastric wall ischemia, perforation, and necrosis. If it is not treated by surgery in time, the mortality rate can range from 30 to 50%. Clinical presentations of acute gastric volvulus are atypical and often mimic other abdominal conditions such as gastritis, gastroesophageal reflux, gastric dilation, and pancreatitis. Imaging studies are crucial for diagnosis, with barium meal fluoroscopy being the primary modality for diagnosing gastric volvulus. Cases of acute gastric volvulus diagnosed by ultrasound are rarely reported.

**Case presentation:**

We reported a rare case of acute gastric volvulus in a 4-year-old Chinese girl who presented with vomiting and abdominal pain. Ultrasound examination revealed the “whirlpool sign” in the cardia region, raising suspicion of gastric volvulus. Diagnosis was confirmed by X-ray barium meal fluoroscopy, which indicated left-sided diaphragmatic hernia and obstruction at the cardia region. Surgical intervention confirmed our suspicion of acute complete gastric volvulus combined with diaphragmatic hernia.

**Conclusion:**

In this case, we reported an instance of acute complete gastric volvulus. Ultrasound revealed a “whirlpool sign” in the cardia, which is likely to be a key sign for the diagnosis of complete gastric volvulus.

## Introduction

Acute complete gastric volvulus is a rare and life-threatening disease in which the stomach rotates more than 180 degrees around its axis [1, 2]. It can lead gastric wall ischemia, perforation, and necrosis. If it is not treated by surgery in time, the mortality rate can range from 30–50% [3]. Clinical presentations of acute gastric volvulus are atypical and often mimic other abdominal conditions such as gastritis, gastroesophageal reflux, gastric dilation, and pancreatitis. Imaging studies are crucial for diagnosis, with barium meal fluoroscopy being the primary modality for diagnosing gastric volvulus. There are limited cases reported of acute gastric volvulus diagnosed by ultrasound. We reported a rare case of acute gastric volvulus in a 4-year-old Chinese girl who presented with vomiting and abdominal pain. Ultrasound examination revealed the “whirlpool sign” at the cardia region, raising suspicion of gastric volvulus. Diagnosis was confirmed by X-ray barium meal fluoroscopy, which indicated left-sided diaphragmatic hernia and obstruction at the cardia region. Surgical intervention confirmed our suspicion. Notably, the patient was unaware of her underlying congenital left diaphragmatic hernia prior to the acute presentation of gastric volvulus. The case highlights the challenge posed by the asymptomatic nature of certain congenital conditions in clinical practice.

## Case description

A 4-year-old girl was admitted to the hospital due to vomiting and abdominal pain that had persisted for two days. Upon admission, clinical examination revealed reduced subcutaneous fat, a soft non-distended abdomen, tenderness around the umbilical region. No other positive findings were observed on physical examination. Routine laboratory tests were all within the normal range.

**Fig. 1:**
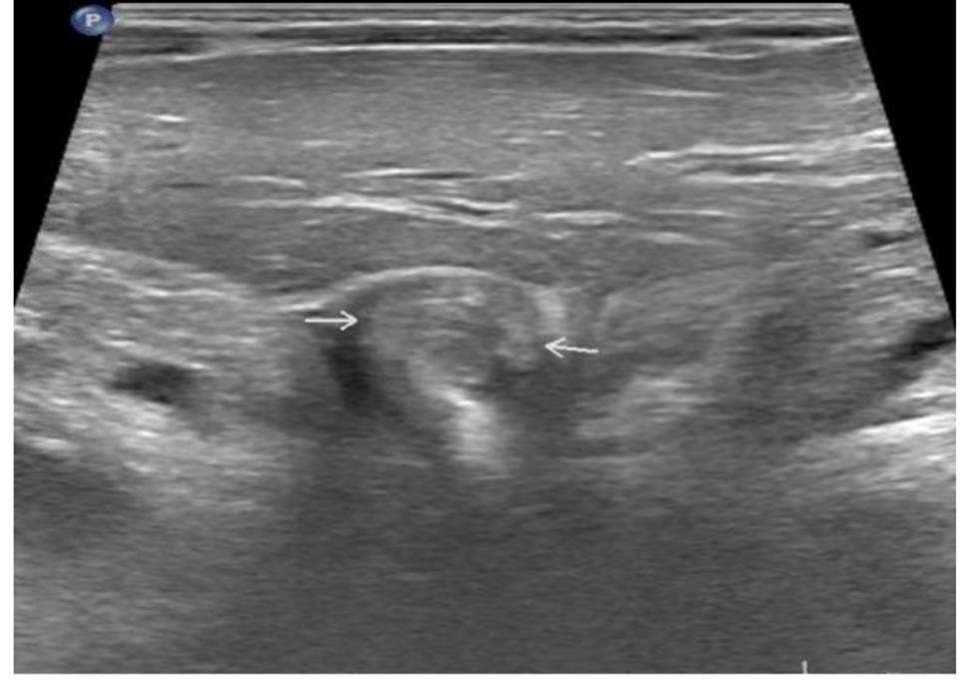
Ultrasound displayed the “whirlpool sign” at the cardia (indicated by the arrow)

**Fig. 2:**
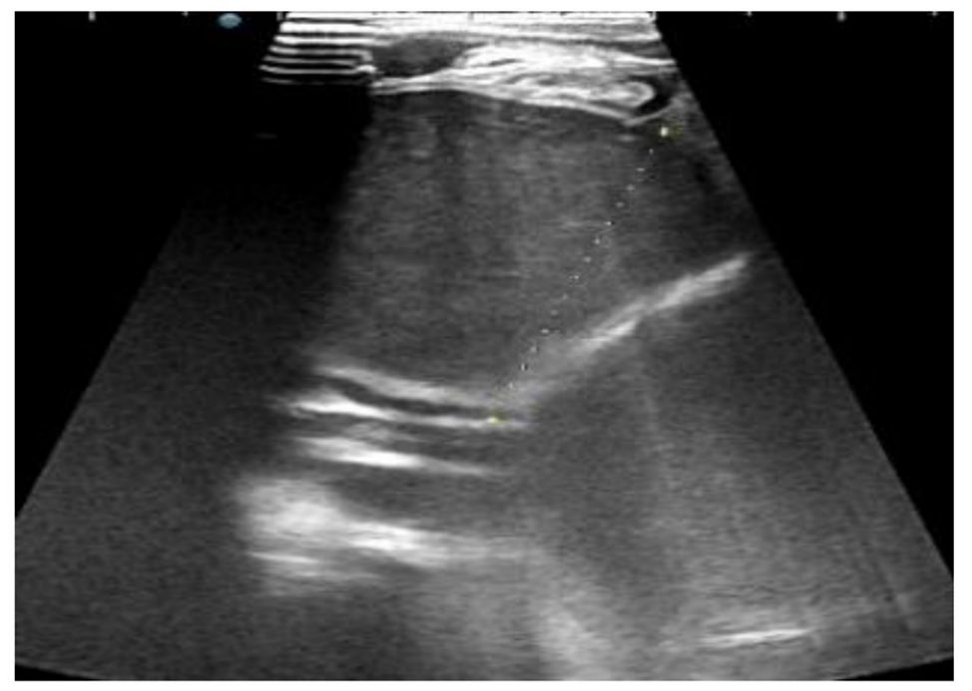
Ultrasound revealed extreme gastric dilation, with a significant portion located within the thoracic cavity

**Fig. 3:**
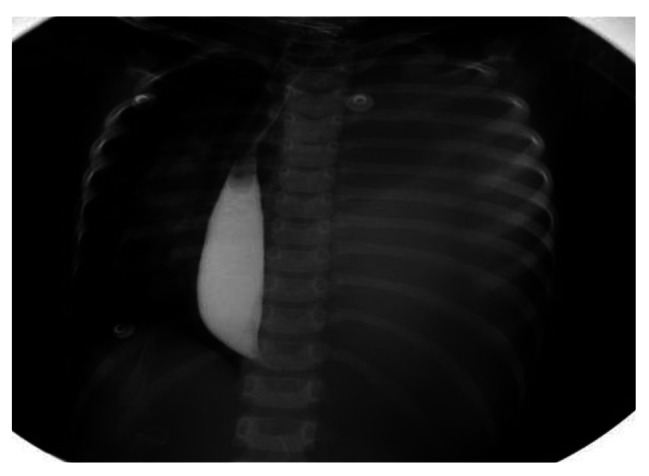
X-ray contrast imaging demonstrated a “bird’s beak” appearance in the distal esophagus

The abdominal plain film showed consistently increased density in the left lung field, absence of a gastric bubble, and an air-fluid level in the lower right abdomen, indicating intestinal obstruction. Abdominal ultrasound examination revealed a “whirlpool sign” approximately 18 × 17 mm^2^ in size at the gastric cardia (Fig. 1) with a counterclockwise rotation. The gastric cavity was dilated, and a portion of the stomach was located within the thoracic cavity (Fig. 2). The pylorus was not clearly visualized. Ultrasound diagnosed: gastric volvulus combined with diaphragmatic hernia. Barium meal fluoroscopy showed a fluid-filled cystic shadow in the left upper to middle lung field that did not significantly change with position. The left diaphragmatic margin was indistinct, the right diaphragmatic margin was smooth, the mediastinum was markedly shifted to the right, and the distal end of the gastric decompression tube was positioned at the level of the right diaphragmatic margin. Following oral administration of a compound barium solution, it was obserevd that the contrast agent filled the esophagus but was obstructed as it descended toward the distal end of the esophagus, resulting in a “bird’s beak” appearance (Fig. 3). The proximal esophagus was dilated, and there was frequent reflux of the contrast agent. Regardless of trying to remove the gastrointestinal decompression tube, the descending of the contrast agent was still blocked. By repeated observations in various positions, the distal digestive tract cannot be visualized. X-ray diagnosed left diaphragmatic hernia and obstruction of the cardia.

**Fig. 4:**
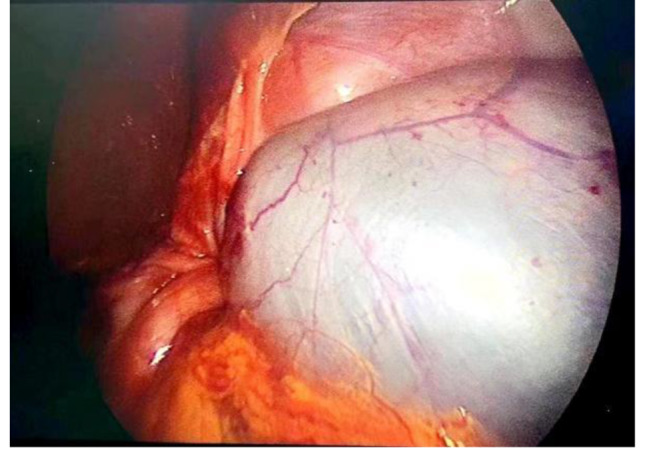
Laparoscopy revealed significant gastric distention

**Fig. 5:**
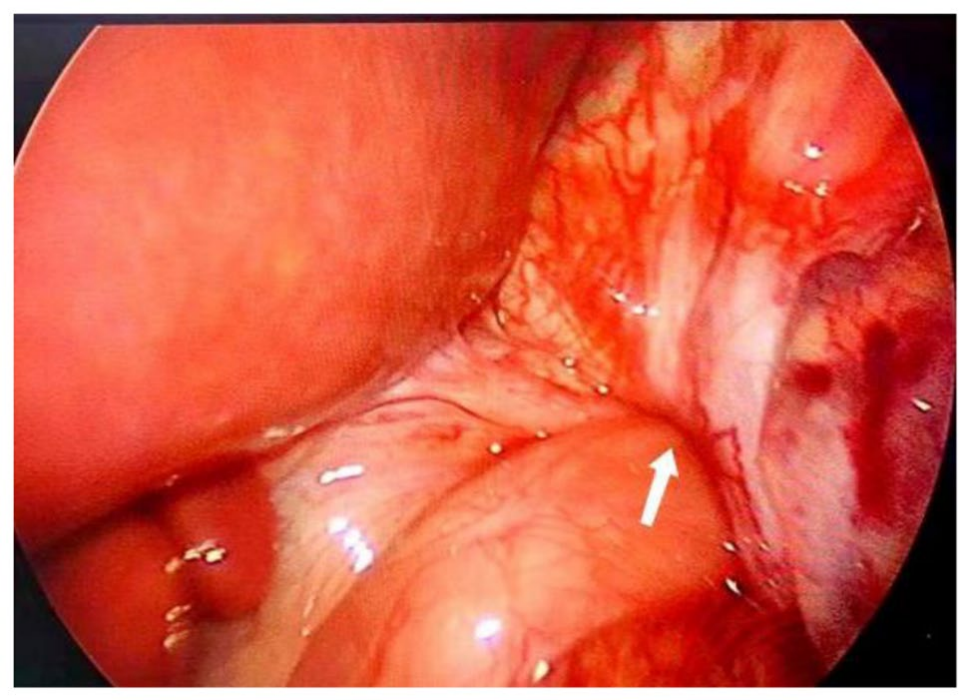
The base of the torsion (indicated by the arrow)

Emergency Surgery and Postoperative Progress: The acute gastric volvulus posed a life-threatening risk to the patient, necessitating emergency surgery. The surgical procedure was as follows: under laparoscopy, a significant defect in the left diaphragm was observed. This diaphragmatic defect existed independently and was not connected to the esophageal hiatus. The stomach, small intestine, spleen, pancreas, and a portion of the colon had all herniated into the left thoracic cavity. The stomach was markedly distended (Fig. 4), exhibiting high tension and local signs of ischemic necrosis. As laparoscopy failed to relocate the abdominal organs to the abdominal cavity, an open surgical approach was adopted. An approximately 8 cm oblique incision below the left costal margin was made to access the abdominal cavity. The stomach, small intestine, spleen, pancreas, and part of the colon were carefully relocated back into the abdominal cavity. During the procedure, it was noted that the stomach had undergone a counterclockwise rotation of 360° (Fig. 5). Following the reduction, areas of ischemic necrosis in the serosal layer of the gastric wall were identified, and repaired by interrupted suturing. The left diaphragm was adequately mobilized, and surrounding adhesions were released. And interrupted sutures with 2 − 0 Micro-bridge sutures were used to repair the diaphragm. Further release of adhesions at the root of the mesentery enhenced reduction. After this, the small intestine and colon were arranged within the abdominal cavity. Gastric fixation was not performed during the surgery. The operation lasted for 3 h and 2 min, with a blood loss of around 52 ml.

Intraoperative Diagnosis: (1) Left diaphragmatic hernia with cardia obstruction, (2) Gastric volvulus. Postoperatively, the patient received mechanical ventilation assistance for 14 h. On the 6th postoperative day, the child’s abdominal drainage fluid increased and turned yellow-green. Bedside ultrasound indicated localized fluid accumulation in the upper left abdomen, suggesting a possible leakage of digestive fluids. An urgent abdominal exploration was performed, which revealed a roughly 3 mm perforation in the gastric wall (at the original suture line). Intermittent full-layer suture were applied to the perforation, and the seromuscular layer was reinforced with suture. The patient managed without respiratory assistance throughout the second surgery. On the 9th postoperative day, the child was gradually allowed to drink water. And the diet was slowly advanced. The patient fully recovered and was discharged 17 days after the surgery. Following discharge from the hospital, we conducted rigorous follow-up procedures. The initial follow-up took place one week post-discharge, followed by monthly check-ins for 8 months. Throughout this period, our focus was primarily on monitoring the patient’s weight and growth. We are pleased to report a weight gain of 2.5 kg and confirm that her growth and development align with normal parameters for her age group during the follow-up period. Despite undergoing two surgeries, the patient’s recovery process was smooth, and she is currently in good health. Long-term follow-up will be continued to ensure her ongoing well-being.

## Discussion

Gastric volvulus refers to an abnormal rotation of the stomach along its long or short axis, leading to gastric obstruction. Gastric volvulus can be categorized as primary or secondary based on its etiology. Primary gastric volvulus occurs when only the gastric ligaments (hepatogastric, gastrohepatic, gastrosplenic, and gastrocolic ligaments) are excessively long, lax, or absent, without the presence of other congenital malformations that might cause the gastric torsion. Secondary gastric volvulus, on the other hand, is caused by abnormalities in adjacent organs such as the diaphragm and/or spleen.

Based on the rotation axis of gastric volvulus, gastric volvulus can be divided into organoaxial, mesenteroaxial, and mixed types [4–6]. The organaxis is the most common type of gastric volvulus, referring to the torsion of the stomach along its long axis (the line between the cardia and the pylorus), and often coexists with a diaphragmatic defect. The mesenteroaxial type refers to the torsion of the stomach along the axis of the midpoint of the great curvature of the stomach and the lesser curvature of the stomach; the mixed type is extremely rare and has both the above two torsion manifestations.

In the presented case, the occurrence of gastric volvulus is related to the congenital diaphragm defect. The position of the stomach is displaced due to the hernia of the stomach through the diaphragm defect into the thoracic cavity or other organs due to ligament pull. The stomach is rotated counterclockwise along the axis of the line between the cardia and the pylorus. Rotation greater than 180° is considered to be complete torsion, which can easily lead to gastric outlet obstruction and strangulation [7–9]. Complete torsion is common in the organoaxial type and is characterized on X-ray fluoroscopy by a sudden narrowing of the lower esophagus and a “bird’s beak” appearance. The case described in our study represents a complete secondary organoaxial gastric volvulus.

Clinically, gastric volvulus can be divided into chronic and acute. Chronic gastric volvulus often presents with intermittent upper abdominal pain, nausea, postprandial vomiting, and abdominal distension. It is typically managed conservatively. Acute gastric volvulus, on the other hand, is not only rarer but has a sudden onset, with severe abdominal pain and non-bilious vomiting as the main symptoms, requiring immediate surgical reduction. The reported case is an instance of acute gastric volvulus, with clinical manifestations of vomiting and abdominal pain. It can be challenging to differentiate acute gastric volvulus from other causes of upper gastrointestinal obstruction. In this particular case, an abdominal X-ray was performed when symptoms appeared, leading to a misdiagnosis of intestinal obstruction.

Acute gastric volvulus is considered a surgical emergency, and if not promptly treated with surgery, it can lead to gastric wall ischemia, perforation, necrosis, and even a life-threatening situation in children. Hence, early diagnosis is of paramount importance. Borchardt described three symptoms to help with the diagnosis: upper abdominal pain, repeated retching, and inability to insert a nasogastric tube [10]. In our case, Borchardt’s triad was prominently exhibited. However, clinical presentations of gastric volvulus in most children are atypical, and some may not exhibit any of the aforementioned clinical symptoms [11, 12]. X-ray fluoroscopy is considered a key diagnostic modality for gastric volvulus [13–15]. However, in cases of complete gastric volvulus, the contrast agent may not pass into the stomach, and the diagnosis may be limited to cardia obstruction. Moreover, there are literatures suggest that barium meal radiography in patients with delayed diagnoses of acute gastric volvulus, especially those with a longer history, may carry a higher risk of gastric perforation [6, 8].

Ultrasound is a non-invasive and convenient method for detecting relevant abdorminal abnormalities such as diaphragmatic hernias, splenic anomalies, and gastric volvulus. It was reported that abdominal ultrasound in an adult patient with chronic intermittent gastric volvulus revealed gastric midportion constriction, referred to as the “peanut sign”, which can be indicative of gastric volvulus [16]. In another study of a 6-year-old patient with acute mesenteroaxial gastric volvulus, ultrasonography highlighted a distended and fluid-filled stomach, which was displaced in a cephalic position compared to esophagus and a pylorus pointing downward in a cranial caudal orientation. The case was ultimately confirmed as gastric volvulus through barium meal fluoroscopy [17].

In this study, we conducted an abdominal ultrasound examination on a patient with acute complete organoaxial gastric volvulus and observed a distinctive feature known as the “whirlpool sign” at the cardia. This sign is a specific sign of torsion-type diseases, and the degree of the whirlpool is closely related to the extent of torsion. In this case, the patient had an exceedingly rare complete gastric volvulus with a 360° rotation, making the “whirlpool sign” highly pronounced. Ultrasound, being a real-time imaging modality that can be performed in any direction, enabled us to clearly visualize the “whirlpool sign” at the cardia. Therefore, this sign proves to be valuable in diagnosing complete gastric volvulus, especially in younger children who may not cooperate with instructions, making ultrasound challenging to X-ray barium studies. Moreover, ultrasound, being non-invasive, painless, and radiation-free, can be repeated as necessary.

In ultrasound examination, the whirlpool sign, as a widely documented finding, can be observed in various intestinal torsions, including small bowel torsion, cecal torsion, sigmoid colon torsion, closed-loop intestinal obstruction, and others [18, 19]. Its significance as a diagnostic indicator is particularly prominent, especially in pediatric patients presenting with acute abdominal pain, where early diagnosis and intervention are crucial. Although gastric torsion is relatively rare, the observation of this feature in ultrasound examinations holds important diagnostic value. In this case report, gastric torsion was successfully diagnosed through ultrasound examination, with the “whirlpool sign” playing a key role in the diagnostic process. Therefore, in similar challenging cases, the use of ultrasound examination should be considered, with a specific focus on the presence of the “whirlpool sign” to enhance opportunities for early diagnosis and treatment.

For acute complete gastric volvulus, immediate surgical intervention is strongly recommended. The choice between laparoscopic and open surgery via the abdominal approach can be based on the surgeon’s preference [20, 21]. The surgical procedure typically involves the following steps: reduction of the gastric volvulus, evaluation of gastric wall, exploration of secondary factors, gastric fixation, and evaluation of the need for anti-reflux operation [17, 22]. In this case, the child underwent laparoscopic surgery to reduce the gastric volvulus and repair the diaphragmatic hernia, but due to the failure of laparoscopy to reduce the abdominal organs back into the abdominal cavity, an open surgical approach was adopted. Gastric fixation was not performed during the surgery.

## Conclusion

Acute complete gastric volvulus is an extremely rare condition in children. Early diagnosis and immediate surgical intervention are key factors for a successful outcome. Our study showed that ultrasound examination plays a valuable role in the diagnosis of acute complete gastric volvulus. In addition to detecting the “whirlpool sign” at the gastroesophageal junction, it can also identify associated anomalies such as hiatal hernia. The “whirlpool sign” on ultrasound is a distinct feature visible in various rotations of the gastrointestinal tract, including gastric volvulus. While acute gastric volvulus itself is uncommon, the presentation of the whirlpool sign is rare. This case further validates the effectiveness of the “whirlpool sign” in another gastrointestinal misalignment—gastric volvulus. The “whirlpool sign” on ultrasound has pathological features, high sensitivity to volvulus, and is a useful indicator. Familiarity with and emphasis on the whirlpool sign during ultrasound examination are crucial for pediatricians and sonographers to ensure timely intervention, thereby improving the prognosis of pediatric patients and reducing the incidence of complications.

## Data Availability

All data generated or analyzed during this study are included in this published article and its supplementary information files.
